# Introducing intravascular microdialysis for continuous lactate monitoring in patients undergoing cardiac surgery: a prospective observational study

**DOI:** 10.1186/cc13808

**Published:** 2014-03-31

**Authors:** Fanny Schierenbeck, Maarten W N Nijsten, Anders Franco-Cereceda, Jan Liska

**Affiliations:** 1 Section of Cardiothoracic Surgery, Department of Molecular Medicine and Surgery, Karolinska Institute, Karolinska University Hospital, S-171 76 Stockholm, Sweden; 2 Department of Critical Care, University of Groningen, University Medical Center Groningen, Hanzeplein 1, NL-9713 GZ Groningen, The Netherlands

## Abstract

**Introduction:**

Lactate is a marker of hypoperfusion and may be used for risk assessment in critically ill patients. Although evidence suggests that repeated lactate measurements are of clinical interest, how and when lactate should be analyzed is controversial. Intravascular microdialysis provides a novel method for the continuous monitoring of lactate, which may be clinically beneficial in critically ill patients.

**Methods:**

Circulating lactate levels were continuously monitored in 80 patients undergoing cardiac surgery using either a separate single-lumen microdialysis catheter or a triple-lumen central venous catheter with an integrated microdialysis function. The catheter was placed with the tip positioned in the superior vena cava. Arterial blood gas samples were taken every hour to obtain reference values, and the lactate levels were analyzed in a blood gas analyzer.

**Results:**

A total of 1,601 paired microdialysis–arterial blood gas lactate samples were obtained. Bland-Altman analysis showed a bias (mean difference) ± limits of agreement (±1.96 SD) of 0.02 ± 0.42 mmol/L. The regression coefficient was 0.98 (*P* = 0.0001).

**Conclusions:**

Central venous microdialysis is an accurate and reliable method for continuous blood lactate monitoring in patients undergoing cardiac surgery. The system may be useful for early lactate-guided therapy in critically ill patients.

## Introduction

Blood lactate level is a clinically important biomarker in critically ill patients, and its measurement may aid in monitoring of hemodynamics [1]. Elevated blood lactate is strongly associated with adverse outcomes in critically ill patients and thus has prognostic value [2-4]. Hyperlactatemia has traditionally been considered to be a sign of tissue hypoperfusion due to anaerobic metabolism [5], such as during cardiogenic shock. Investigators in recent studies have suggested that elevated blood lactate during critical disease is the result of not only tissue hypoperfusion but also adrenergic stress [6]. This phenomenon was demonstrated for skeletal muscle, where epinephrine activates the Na^+^/K^+^-ATPase pump independently of tissue hypoxia [7]. An elevated blood lactate concentration may result from both increased production and decreased clearance. Lactate clearance occurs mainly in the liver and is dependent on tissue perfusion. In addition to elevations in lactate, the rate of normalization of elevated lactate levels is also independently associated with increased mortality [8,9].

Lactate monitoring may thus be used for risk stratification in critically ill patients [10,11] and for early lactate-guided treatment. Researchers in a prospective randomized trial showed that lactate-guided treatment in patients with hyperlactatemia resulted in improved outcomes [12]. Cardiac surgery patients often develop hyperlactatemia postoperatively [13], which may be affected by several factors, such as the type of surgery, patient body temperature and extracorporeal circulation. Elevated lactate levels in this patient category are also associated with postoperative mortality and a prolonged ICU stay [14,15]. A similar relation was observed for pediatric cardiac surgery patients [16].

We have previously evaluated intravascular microdialysis to monitor glucose, both offline and continuously [17-19]. In our first study, we compared intravascular microdialysis lactate values offline with arterial blood lactate concentration [17], but we did not study the online continuous monitoring of lactate. The aim of our present observational study was to evaluate intravascular microdialysis as a method for continuous lactate monitoring in cardiac surgery patients by comparing the accuracy of microdialysis lactate values with reference values analyzed by an arterial blood gas test. In addition, we recorded major postoperative complications in relation to lactate levels.

## Methods

### Ethical approval

The study was approved by the Stockholm Regional Ethical Review Board. Before their inclusion in the study, patients gave their written consent to participate.

### Patients

The study included 80 randomly selected patients who underwent routine elective cardiac cardiopulmonary bypass surgery at the Karolinska University Hospital in Stockholm during two time periods: between March and July 2010 (*n* = 50 patients monitored with a single-lumen catheter (SLC)) and between May and August 2011 (*n* = 30 patients monitored with a triple-lumen catheter (TLC)). The study groups were separated in time because different microdialysis catheters were used (see patient monitoring). However, the microdialysis equipment used was the same in both groups (microdialysis-membrane, sensor and monitor system). We previously evaluated intravascular microdialysis for continuous glucose monitoring in patients monitored with the SLC [18] and patients monitored with the TLC [19].

### Patient monitoring

**Figure 1:**
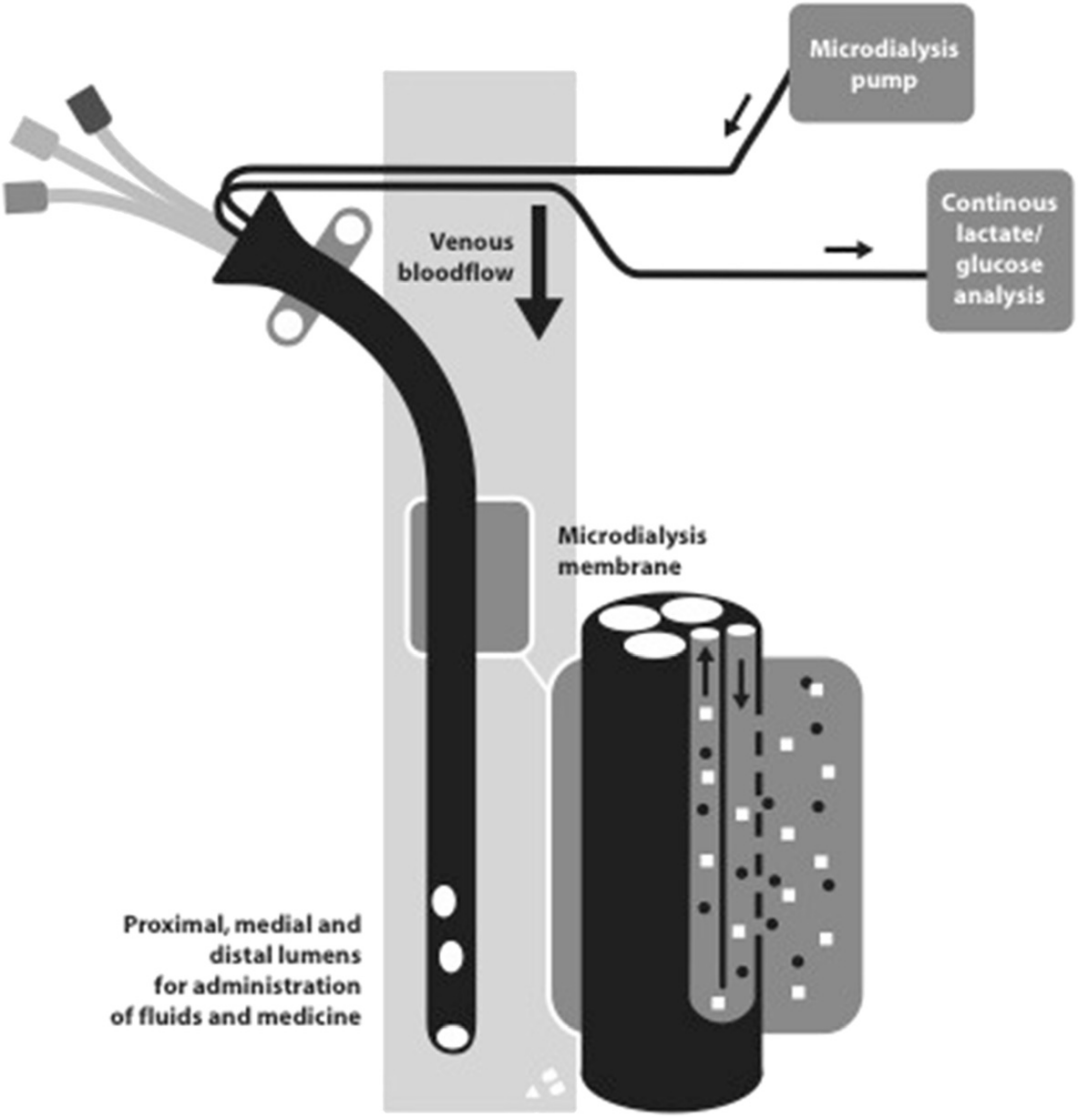
**Schematic illustration of the intravascular microdialysis system.** The microdialysis membrane is placed in the bloodstream and is continuously perfused with a perfusion fluid, creating a dialysate. Due to the diffusion of small molecules (for example, lactate) over the microdialysis membrane, the concentration of these molecules in the dialysate will be the same as that in the bloodstream. The dialysate is then analyzed by a sensor that converts this information to digital signals, resulting in a numerical value and a trend graph of the lactate values on a monitor.

Blood lactate was continuously monitored in all patients using the Eirus intravascular microdialysis system (Maquet Critical Care, Solna, Sweden), which measures small metabolites such as glucose and lactate without blood sampling. The first group of patients (*n* = 50) had a separate single-lumen microdialysis catheter for microdialysis (Eirus SLC; Maquet Critical Care), and the second group of patients (*n* = 30) had a TLC (Eirus TLC; Maquet Critical Care) functioning as a regular central venous catheter (CVC) with an integrated microdialysis function. The patients monitored with a SLC had an additional CVC for sufficient central venous access. Both the SLC and the TLC were connected to a sensor that continuously analyzes glucose and lactate values and displays them on a screen. A schematic illustration of the microdialysis system is provided in Figure 1. All the patients had the microdialysis catheter placed preoperatively after being put under general anesthesia. The catheter was inserted into the internal jugular vein as a regular CVC. The length of the SLC is 30 cm, and the length of the TLC is 16 cm. Both catheters were positioned with placement of the microdialysis membrane in the superior vena cava/right atrium. Thus the TLC was inserted for its whole length, whereas the SLC was inserted according to length markers on the catheter. The microdialysis catheter was then connected to the sensor, and the perfusion of the system was started with 0.9% sodium chloride. The perfusion of the system takes approximately 5 minutes, implying a time lag between the actual lactate levels present near the microdialysis membrane and the levels measured *ex vivo* in the dialysis fluid. The Eirus system recorded and displayed the lactate values.

In addition to enabling the microdialysis system, the TLC was used for both drug administration and blood sampling and was removed when a central line was no longer necessary. The SLC was removed when the continuous lactate monitoring was ended. After removal, the microdialysis membrane was checked for potential blood clotting. The patients were given standard antithrombotic prophylaxis with low-molecular-weight heparin (LMWH) 24 hours after surgery. Patients with an indication for warfarin were started on LMWH until a therapeutic international normalized ratio was reached.

### Measurements

Arterial blood gases were taken as reference values once every hour and were analyzed in a blood gas analyzer (ABL800 FLEX; Radiometer Medical, Copenhagen, Denmark). The lactate values displayed by the microdialysis system were then compared with these reference values. The microdialysis system is calibrated every 8 hours using the reference arterial blood gas. The calibrated values were not included for comparison. All other paired microdialysis–arterial blood gas values were included for analysis. For the optimal evaluation of the microdialysis-generated results, the arterial blood gas values were compared with the microdialysis results obtained after the 5-minute time lag. The monitoring period was ceased when the patient was discharged from the ICU or after 48 hours. However, patients monitored with the TLC had the catheter in place as long as a central line was necessary and single paired measurements could be obtained after the 48-hour monitoring period at random times.

### Statistical analysis

Bland-Altman analysis was performed using MedCalc version 12.7.5 statistical software (MedCalc Software, Ostend, Belgium), adjusting for the fact that multiple paired samples were obtained from each patient. Bland-Altman analysis displays the bias (mean of differences) and limits of agreement (bias ± 1.96 SD) of the paired lactate values. Overall association between microdialysis and arterial blood gas lactate concentrations was estimated using a regression coefficient, which was calculated using a random coefficient mixed model in SAS version 9.3 statistical software (SAS Institute, Cary, NC, USA). Remaining statistical analyses, including Pearson correlation coefficients for each patient, were carried out using IBM SPSS version 22 statistical software (IBM SPSS, Chicago, IL, USA). If a high lactate level (>3 mmol/L) was measured, the possible association with a clinical outcome was investigated.

## Results

Data were available for 78 patients. Data were missing for two patients monitored with the SLC due to catheter damage that occurred while performing mitral valve surgery in one patient and due to mechanical sensor failure in the other. Data were available for analysis in all patients monitored with the TLC. However, monitoring was ended prematurely in one patient because of catheter dislocation (accidental withdrawal), which was subsequently detected as the microdialysis values were accompanied by a warning signal due to rapid changes in blood lactate levels. In three other patients, the monitoring was paused because of sensor complications, which were also detected by the accompanying warning signal for uncertain values, but could be restarted after exchange of the sensor. Two patients monitored with the TLC had shorter follow-up times than expected because, by accident, monitoring was not conducted during nighttime. The patient characteristics from the included 78 patients are shown in Table 1. The mean follow-up time for all patients was 25.6 hours (range = 1 to 97, median = 22), and a total of 1,601 paired microdialysis–arterial blood gas lactate values were obtained and included for analysis. An average of 20.5 samples from each patient (range = 1 to 43, median = 19) were analyzed. The values for each patient are provided in Table 2. No consistent systematic drift between calibrations was observed (see Additional file 1). Furthermore, we did not notice any increasing calibration drift with longer monitoring time over subsequent calibration periods (see Additional file 2). After the removal of the microdialysis catheter, no blood clotting of the microdialysis membrane was observed and no complications caused by the microdialysis catheter were detected.

**Table 1:** **Patient characteristics**^**a**^

**Characteristics**		**Count**	**Median**	**Interquartile range**
Sex	Female	22		
	Male	56		
Age (years)			69	14
BMI			25.5	5.35
Ejection fraction (%)			50	10
Diabetic status	No diabetes	57		
	Diabetes type 1	2		
	Diabetes type 2	19		
Surgical procedure	CABG	33		
	AVR	24		
	AVR + CABG	7		
	MVR	2		
	MVR + CABG	1		
	Aortic graft	4		
	AVR + aortic graft	5		
	Other	2		
Time of extracorporeal circulation (min)			78	53
Time of aortic cross-clamping (min)			56	47
Vasoactive drugs	None	4		
	NA	9		
	Nitro	30		
	NA + Nitro	29		
	NA + Nitro + A	1		
	NA + Nitro + Milrinone	1		
	NA + Nitro + Levosimendan	1		
	NA + Milrinone	1		
	NA + Milrinone + A	1		
	NA + Milrinone + Levosimendan	1		
Transfusions	Red blood cells	44	2	4
	Plasma	16	0	1

^a^A = Adrenalin, AVR = Aortic valve replacement, BMI = Body mass index, CABG = Coronary artery bypass grafting, MVR = Mitral valve replacement, NA = Noradrenalin, Nitro = Nitroglycerin. Information is provided as either count (number of patients) or median and interquartile range. For transfusions both the number of patients receiving either plasma or red blood cell transfusion as well as the median and interquartile range of the number of transfusions administered are presented.

**Table 2:** **Microdialysis–arterial blood gas lactate paired samples data per patient**^**a**^

**Catheter**	**Patient**	**Number of paired samples per patient**	**Correlation coefficient ( *r *)**	***P*-value**
SLC	1	20	0.98	0.0001
SLC	3	18	0.904	0.0001
SLC	4	15	0.931	0.0001
SLC	5	20	0.948	0.0001
SLC	6	18	0.817	0.0001
SLC	7	16	0.896	0.0001
SLC	8	18	0.94	0.0001
SLC	9	20	0.947	0.0001
SLC	10	39	0.889	0.0001
SLC	11	17	0.952	0.0001
SLC	12	19	0.985	0.0001
SLC	13	9	0.949	0.0001
SLC	14	10	0.954	0.0001
SLC	15	18	0.835	0.0001
SLC	16	17	0.971	0.0001
SLC	17	41	0.959	0.0001
SLC	18	17	0.896	0.0001
SLC	19	35	0.982	0.0001
SLC	20	17	0.401	0.11
SLC	21	39	0.753	0.0001
SLC	22	20	0.681	0.001
SLC	23	39	0.884	0.0001
SLC	24	19	0.738	0.0001
SLC	25	20	0.804	0.0001
SLC	26	21	0.966	0.0001
SLC	27	29	0.95	0.0001
SLC	28	18	0.98	0.0001
SLC	29	20	0.939	0.0001
SLC	30	20	0.93	0.0001
SLC	32	19	0.989	0.0001
SLC	33	9	0.704	0.034
SLC	34	8	0.96	0.0001
SLC	35	17	0.99	0.0001
SLC	36	21	0.978	0.0001
SLC	37	19	0.908	0.0001
SLC	38	38	0.995	0.0001
SLC	39	21	0.935	0.0001
SLC	40	21	0.926	0.0001
SLC	41	19	0.956	0.0001
SLC	42	18	0.875	0.0001
SLC	43	18	0.939	0.0001
SLC	44	21	0.87	0.0001
SLC	45	20	0.949	0.0001
SLC	46	16	0.937	0.0001
SLC	47	19	0.939	0.0001
SLC	48	21	0.974	0.0001
SLC	49	20	0.977	0.0001
SLC	50	20	0.88	0.0001
TLC	1	19	0.945	0.0001
TLC	2	34	0.903	0.0001
TLC	3	19	0.846	0.0001
TLC	4	18	0.573	0.013
TLC	5	5	0.811	0.096
TLC	6	19	0.474	0.04
TLC	7	10	0.993	0.0001
TLC	8	20	0.974	0.0001
TLC	9	18	0.886	0.0001
TLC	10	24	0.927	0.0001
TLC	11	20	0.972	0.0001
TLC	12	1	–	–
TLC	13	36	0.956	0.0001
TLC	14	20	0.979	0.0001
TLC	15	19	0.971	0.0001
TLC	16	21	0.976	0.0001
TLC	17	22	0.987	0.0001
TLC	18	41	0.929	0.0001
TLC	19	20	0.987	0.0001
TLC	20	43	0.957	0.0001
TLC	21	2	1	0.0001
TLC	22	19	0.19	0.436
TLC	23	21	0.95	0.0001
TLC	24	43	0.985	0.0001
TLC	25	15	0.522	0.046
TLC	26	20	0.781	0.0001
TLC	27	21	0.992	0.0001
TLC	28	10	0.867	0.001
TLC	29	21	0.968	0.0001
TLC	30	6	0.833	0.039

^a^SLC, Single-lumen catheter; TLC, Triple-lumen catheter.

**Figure 2:**
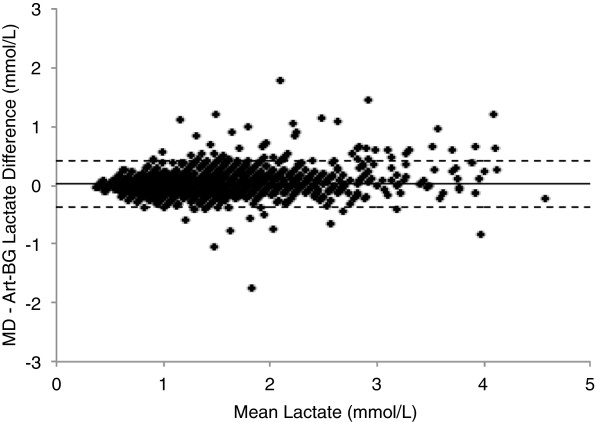
**Scatterplot representing Bland-Altman analysis.** Mean microdialysis lactate and reference arterial blood gas lactate values vs. the difference between the two statistical analysis methods. Bold line represents bias (mean difference), and dotted lines represent limits of agreement (±1.96 SD). Art-BG, Arterial blood gas; MD, Microdialysis.

**Figure 3:**
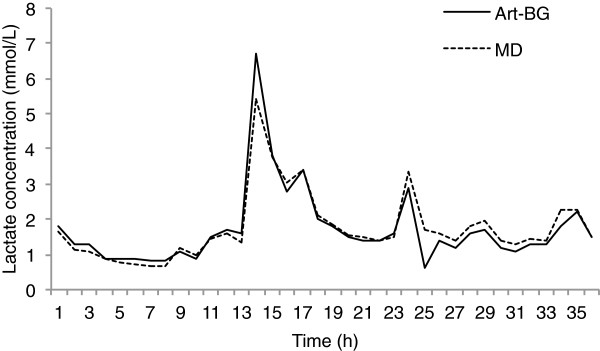
**Example of one patient’s microdialysis and arterial blood gas lactate values over time.** This patient had a maximal microdialysis blood lactate value of 5.4 mmol/L while experiencing postoperative complications with circulatory instability and transient visceral ischemia. Art-BG, = Arterial blood gas; MD, Microdialysis.

The mean (±SD) microdialysis lactate value was 1.40 ± 0.72 mmol/L, and the mean arterial blood gas lactate value was 1.38 ± 0.68 mmol/L. The regression coefficient (calculated using a random coefficient model) was 0.98 (*P* = 0.0001). The Pearson correlation coefficients (*r*) for each patient are presented in Table 2 (median = 0.92, range = 0.19 to 1). The mean relative difference was 1.5%, and the mean absolute relative difference was 10.3%. The Bland-Altman analysis showed a bias ± limits of agreement of 0.02 ± 0.42 mmol/L (Figure 2). An example of a patient’s microdialysis lactate values compared with arterial blood gas lactate values is shown in Figure 3.

In 18 patients, a microdialysis lactate value >3.0 mmol/L was obtained, and the following adverse events occurred in seven of these patients: reoperation for bleeding (*n* = 5), perioperative myocardial infarction (*n* = 1) and circulatory instability with transient visceral ischemia (*n* = 1). If the cutoff point had been set to 2.5 mmol/L, another patient who underwent reoperation for bleeding would have been noted, as would another nine patients for whom no adverse events were recorded. A cutoff point <2.5 mmol/L would not have added more patients with recorded adverse events.

## Discussion

In this study, we demonstrate that intravascular microdialysis may be used for the continuous monitoring of lactate in patients undergoing cardiac surgery. The microdialysis lactate values were well correlated and had low bias compared with reference arterial blood gas lactate values.

The system recognized its malfunction in six patients (as described in Results, first paragraph) and could be corrected for, and the system restarted in three patients. Overall, the correlation coefficient for each patient showed good association between microdialysis lactate and arterial blood gas lactate concentrations. However, a few patients with low correlation coefficients need to be discussed. The reason for correlation coefficients <0.5 was one or two outliers. For example, patient 6, who was monitored with the TLC, the correlation coefficient would be 0.83 (*P* = 0.0001) instead of 0.19 if one outlier were removed. Similarly, removal of one or two outliers in the remaining patients with correlation coefficients <0.5 would yield new correlation coefficients >0.75 (all with *P* < 0.01).

The monitoring of lactate has been made easier with point-of-care testing. Studies have been conducted to analyze the differences in arterial, venous and capillary lactate monitoring methods with findings of high accuracy [20]. There are several reasons why we used arterial samples both as a reference measurement method and for calibration of the central venous microdialysis measurements. First, arterial samples of lactate (and glucose) may be considered the standard method, and they are regularly taken in critically ill patients as opposed to central venous samples. Differences between central venous and arterial lactate concentrations are usually minimal [21,22]. Weil *et al*. studied the correlation between central venous and arterial lactate values and observed a mean difference of 0.03 mmol/L (*r* = 0.996) [23]. In our previous study, we also found good agreement between arterial and venous blood gas lactate values (mean difference = 0.09 mmol/L, limit of agreement ±0.6 mmol/L and Pearson correlation coefficient = 0.92), further supporting arterial blood gas as a reference method [17].

Lactate monitoring in critically ill patients is preferably performed by assessing the lactate trend by repeated lactate sampling, which may be used for predicting in-hospital mortality [11]. Jansen and colleagues used a time interval of 2 hours in their early lactate-guided therapy study [12]. Continuous monitoring may be advantageous for the critically ill patient, as the clinician is provided information every minute that could potentially be used to support and evaluate the given treatment.

Interestingly, we observed a higher complication rate in patients with a postoperative lactate level >3 mmol/L. It should be stressed that the goal of our study was not to evaluate lactate-guided treatment. The sample size was not adequate to evaluate the association between lactate concentration and outcome. However, this observation has some value because continuous lactate monitoring may add further knowledge in this field, which is why we mention this observation in our results. The hypothetical cutoff point of >3.0 mmol/L was based on a recent study in which the researchers reported the association between high lactate values and postoperative complications in adult cardiac surgery patients [15]. Investigators in other studies in which a higher cutoff point (3.0 to 3.5 mmol/L) was used have reported a better predictive value relating hyperlactatemia with mortality [2], even though significantly increased mortality has previously been observed with a cutoff point of 1.5 mmol/L [24].

We hypothesize that the continuous monitoring of lactate may aid in lactate-guided treatment in patients who present with elevated lactate levels, but this remains to be further studied. Studies on lactate-guided treatment have been performed early in critical illness (set to the first 8 hours after ICU admission in the study by Jansen *et al*. [12]), and thus it seems to be important to start lactate monitoring as early as possible. Hence, eligible patients should receive the TLC with integrated microdialysis function as soon as possible after arrival to the ICU. This protocol should not constitute a clinical problem, because these patients usually require rapid central venous access and thus could just as easily receive the microdialysis TLC in place of a regular CVC. If a clinical benefit could be demonstrated with the continuous monitoring of lactate, the cost-effectiveness of utilizing TLCs instead of CVCs would need to be calculated as well.

One should be mindful that this is a pilot study that needs to be further substantiated. In our present study, intravascular microdialysis was evaluated for continuous lactate monitoring in patients undergoing cardiac surgery and not in other patient categories. Additional studies of intravascular microdialysis in surgical and medical ICU patients would be of value.

## Conclusions

We demonstrate that central venous microdialysis is a robust method for continuous blood lactate monitoring in patients undergoing cardiac surgery. This system constitutes a novel and interesting new method of monitoring blood lactate concentration and may be useful in monitoring critically ill patients.

## Key messages

• Persistent elevated lactate concentration in critically ill patients is associated with adverse outcomes.

• Continuous monitoring of lactate may be performed using intravascular microdialysis.

• Intravascular microdialysis lactate values correlated well with arterial blood gas reference measurements.

• Continuous lactate monitoring enables lactate-guided therapy.

## Abbreviations

A: Adrenalin; Art-BG: Arterial blood gas; AVR: Aortic valve replacement; BMI: Body mass index; CABG: Coronary artery bypass graft; CVC: Central venous catheter; CVP: Central venous pressure; LMWH: Low-molecular-weight heparin; MD: Microdialysis; MVR: Mitral valve replacement; NA: Noradrenalin; Nitro: Nitroglycerin; SLC: Single-lumen catheter; TLC: Triple-lumen catheter.

## Competing interests

JL and AFC are minor shareholders in Dipylon Medical AB. The remaining authors declare that they have no competing interests.

## Authors’ contributions

FS analyzed all the data and drafted the manuscript. JL and AFC set up the study design and helped with data analysis and manuscript revision. MWN aided in the study design and manuscript revision. All authors read and approved the final manuscript.

## Supplementary Material

Additional file 1 **Graph depicting lactate calibration drift.** No systematic drift between calibrations could be detected by plotting the difference in lactate (microdialysis lactate–arterial blood gas lactate (MD–ArtBG)) in millimolar concentrations per liter against time after calibration. Click here for file

Additional file 2 **Scatterplot of lactate differences over monitoring time.** We did not observe an increasing calibration drift with longer monitoring time over subsequent calibration periods, as shown by plotting the difference in lactate (microdialysis lactate–arterial blood gas lactate (MD–ArtBG)) in millimolar concentrations per liter against follow-up time. Click here for file
