# Supplementary figures and images for: Introducing intravascular microdialysis for continuous lactate monitoring in patients undergoing cardiac surgery: a prospective observational study
Source: Crit Care. 2014 Mar 31;18(2):R56. doi: 10.1186/cc13808 (PMC4057446; doi:10.1186/cc13808)

Lactate calibration drift

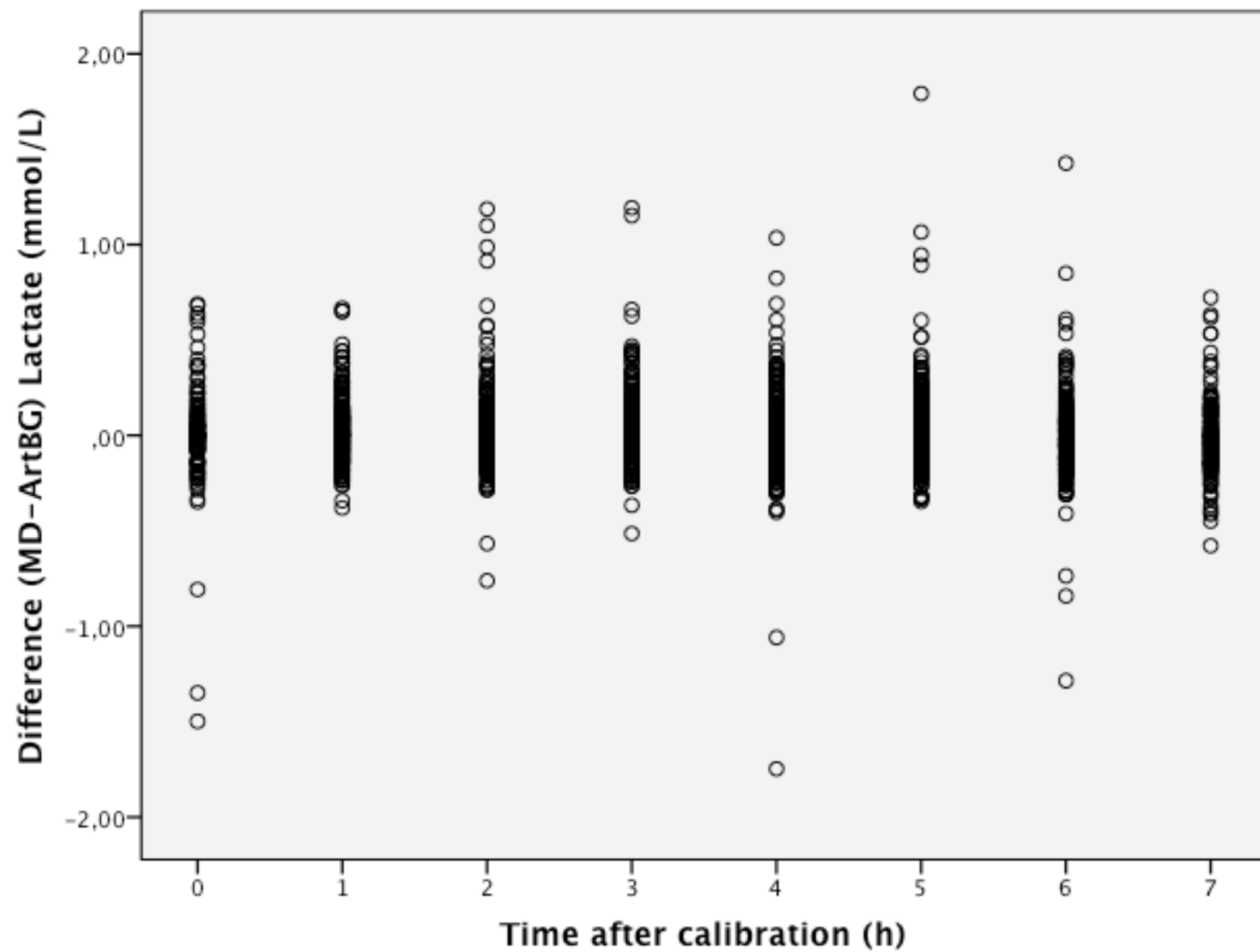

Supplement: Additional file 1 — Graph depicting lactate calibration drift. No systematic drift between calibrations could be detected by plotting the difference in lactate (microdialysis lactate–arterial blood gas lactate (MD–ArtBG)) in millimolar concentrations per liter against time after calibration. [file cc13808-S1.pdf]

**Lactate difference over monitoring time**

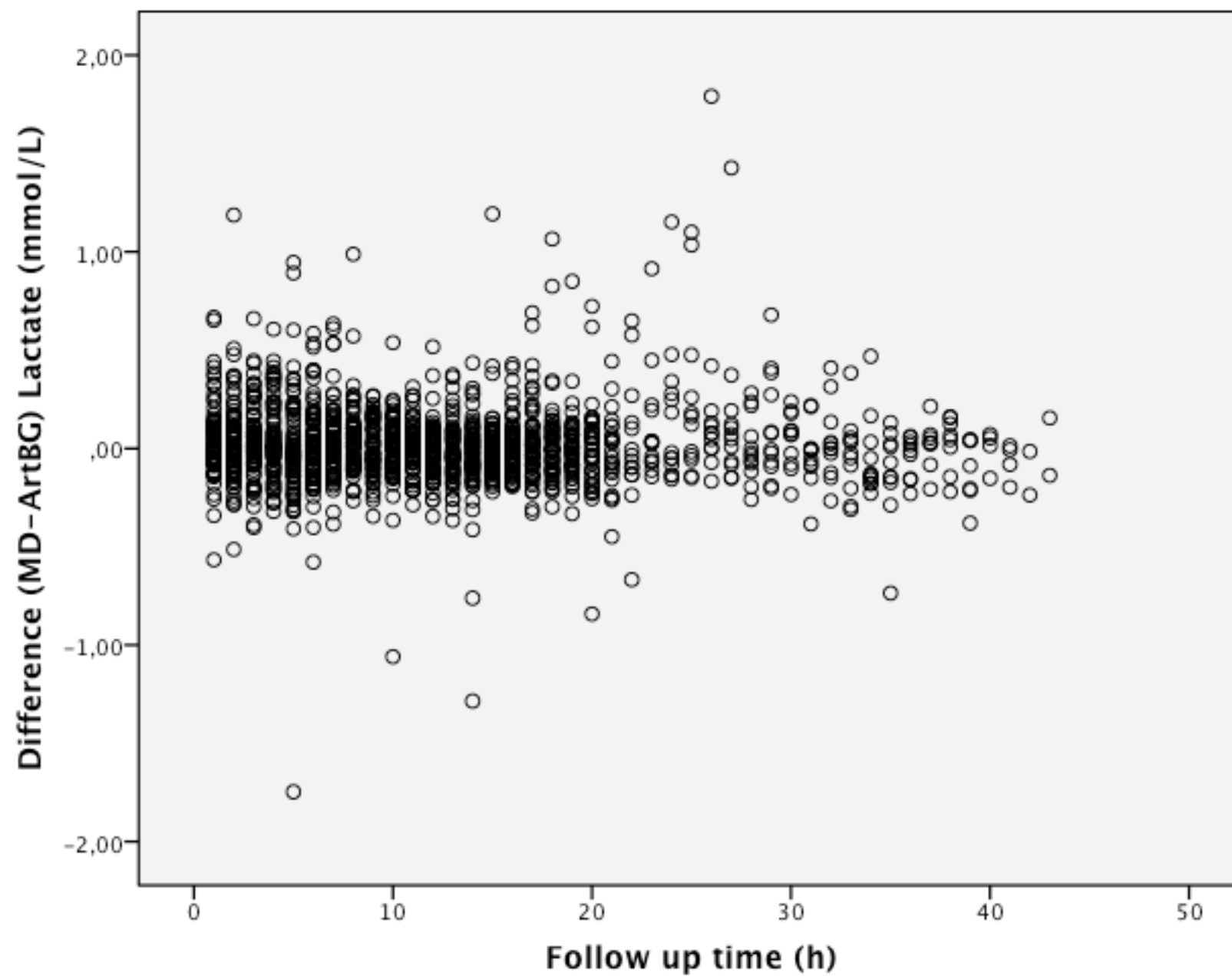

Supplement: Additional file 2 — Scatterplot of lactate differences over monitoring time. We did not observe an increasing calibration drift with longer monitoring time over subsequent calibration periods, as shown by plotting the difference in lactate (microdialysis lactate–arterial blood gas lactate (MD–ArtBG)) in millimolar concentrations per liter against follow-up time. [file cc13808-S2.pdf]
